# Stable isotope ratio analysis: an emerging tool to trace the origin of falsified medicines

**DOI:** 10.1016/j.trac.2024.117666

**Published:** 2024-05

**Authors:** Alberto Roncone, Simon D Kelly, Zoe Giannioti, Cathrin Hauk, Céline Caillet, Paul N Newton, Carla Perez-Mon, Luana Bontempo

**Affiliations:** a Traceability Unit, Research and Innovation Centre, Fondazione Edmund Mach, Via E. Mach 1, 38098, San Michele All’Adige, TN, Italy; b Food Safety & Control Laboratory, Joint FAO/ IAEA Centre of Nuclear Techniques in Food and Agriculture, International Atomic Energy Agency, Vienna International Centre, Wagramer Strasse 5, P.O. Box 100, 1400, Vienna, Austria; c NDM Centre for Global Health Research, Centre for Tropical Medicine and Global Health, Nuffield Department of Medicine, University of Oxford, Oxford, United Kingdom; d Medicine Quality Research Group, Centre for Tropical Medicine and Global Health, Nuffield Department of Medicine, University of Oxford, Oxford, United Kingdom; e Infectious Diseases Data Observatory, Centre for Tropical Medicine and Global Health, Nuffield Department of Medicine, University of Oxford, Oxford, United Kingdom; f Mahidol Oxford Tropical Medicine Research Unit, Faculty of Tropical Medicine, Mahidol University, Bangkok, Thailand; g Royal (Dick) School of Veterinary Studies and the Roslin Institute, University of Edinburgh, Midlothian, EH25 9RG, United Kingdom

## Abstract

Falsified medicines pose a serious threat to global public health. Over the past few decades, the number of public health issues and seizures of falsified medicines has dramatically increased across the world. The development of new analytical techniques for the identification and traceability of these products hold great promise for innovation to help curtail the high number of deaths caused by the lack of adequate treatments and in combating the criminals responsible for manufacturing these products. This review presents the main approaches, based on stable isotope ratios of the bio-elements, mainly Isotope Ratio Mass Spectrometry and Site-specific Natural Isotopic Fractionation by Nuclear Magnetic Resonance, that can contribute to identifying the origin of these products, both in terms of geographical origin and raw materials employed as well as for the batch controls by the producers.

## 1 Introduction

### 1.1 Falsified and substandard pharmaceuticals

Over the past decades, increasing public health-related issues have been reported globally due to falsified and substandard medical products. This phenomenon is causing significant morbidity and mortality, whilst simultaneously reducing the effectiveness of health care and potentially reducing public trust in the effectiveness of medicines and health care systems [1,2].

Substandard medicines are the result of poor manufacturing practices, inappropriate formulation, packaging or storage of the medicine that can lead to deterioration of the product [2,3].

In contrast, the World Health Organization (WHO) defines falsified (aka counterfeit) products as [4]:”Medical products that deliberately/-fraudulently misrepresent their identity, composition or source.” This includes medicines with the correct or incorrect active pharmaceutical ingredient(s) (APIs); without API, with insufficient API(s) or with manipulated packaging or packaging information. Visual inspection and pharmacological analysis may not be sufficient to make a clear distinction between substandard and falsified products and there are few laboratory tools to estimate where falsified medicines have come from [5]. Stable isotope ratio analysis could make an important contribution to facilitate clear differentiations between these two types of poor-quality medicine and estimate their origin.

Substandard and falsified (SF) medicines are a public health threat and can have a severe impact on public health as well as economic and socioeconomic consequences [2,6]. Delayed, incomplete, or lack of recovery are possible consequences of the absence or insufficient amount of the stated API or poor dissolution. In addition, toxic ingredients that may be present in these products can harm patients (i.e. diethylene glycol in cough syrups [7]) and SF antimicrobials may lead to the development and spread of antimicrobial resistance (AMR) [8].

According to WHO, 1 in 10 medical products circulating in low- and middle-income countries (LMICs) are estimated to be substandard or falsified [9]. Reports of all types of medical products can be found in the WHO Global Surveillance and Monitoring System for substandard and falsified medical products (GSMS) portal. However, anti-infectives seem to be the most afflicted therapeutic class [2]. Estimates suggest that this leads every year to the death of 2000 to 72,000 children with pneumonia worldwide and to the death of 31,000 to 116,000 patients from malaria in Sub-Saharan Africa alone [6].

Although this is a global issue, the burden of SF medicines is disproportionately higher in LMIC. Constrained access, weak technical capacities e.g. in national quality control laboratories and poor medicine regulation are the main factors that contribute to a high prevalence of falsified medicines [2].

Frequently, the high prices of medicinal products and the high demand for pharmaceuticals exceeding supply forces people to buy medicines from unauthorised sellers. Easier access and lower prices are among the most influential justifications for using informal markets, but there are also cultural, social and religious reasons[2].

Porous borders, poorly regulated supply chains of medical products, low penalties for the production and trade in falsified medicines, as well as a lack of cross-country control and collaboration makes it difficult to tackle this problem [10]. From a financial perspective, according to the European Federation of Pharmaceutical Industries Association (EFPIA), the trade in falsified pharmaceuticals is more profitable than with illicit drugs, based on an estimated world trade of about $75 billion dollars/year [11]. Other estimates suggest that the trade with falsified products is worth $200 billion dollars [12].

### 1.2 Falsified medical product proliferation

**Fig. 1:**
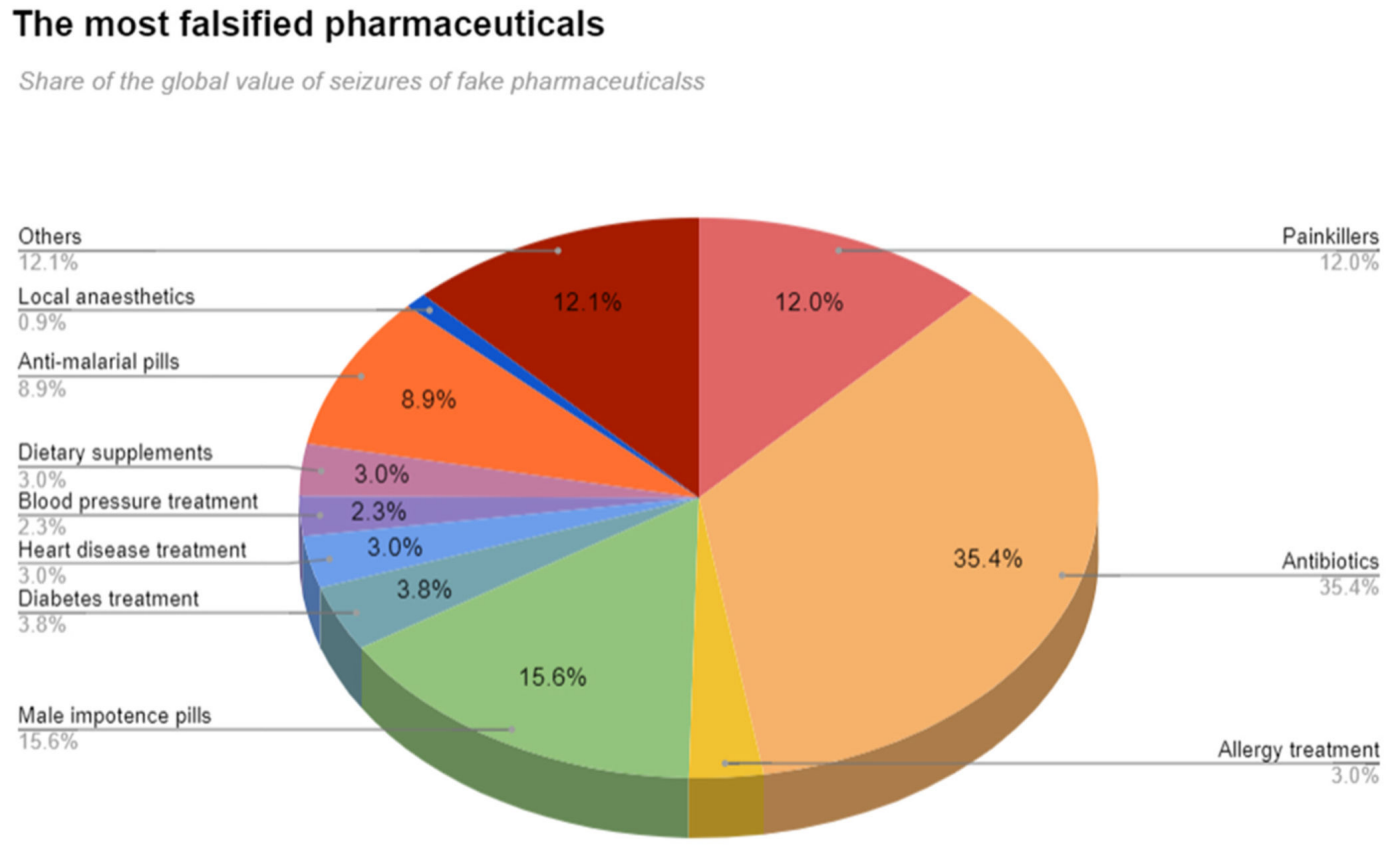
Most counterfeited pharmaceuticals. Source: OECD-EUIPO [13].

Between 2014 and 2016 the Organisation for Economic Co-operation and Development (OECD) reported that antibiotics, male impotence (erectile dysfunction) pills and painkillers are the most frequently falsified medicines seized worldwide Fig. 1.

The global falsified medical product trade is thriving particularly across Africa, in particular for contraceptive pills, cough syrups, antibiotics (especially ‘older’ antibiotics, such as penicillin, tetracycline, trimethoprim–sulfamethoxazole and chloramphenicol), anti-parasitics, and antimalarials are the most common products seized by the law enforcement agencies [14–16].

Due to the increasing popularity of buying medicines online, countries with a well-regulated supply chain are also affected by falsified medicines sold through illegal online pharmacies, including both the USA and Europe. According to Europol, in these regions, the most falsified medicines are lifestyle drugs such as sildenafil (Viagra®) or weight-loss drugs and those for chronic conditions [1,17].

On December 14, 2021, Europol published a report on a large, co-ordinated operation against falsified pharmaceuticals and doping named Shield II, involving law enforcement authorities from 26 countries (20 EU Member States and 7 third-party countries). Officers targeted organised criminal groups trafficking doping substances (hormone and metabolic regulators) and various medications such as anti-cancer drugs, erectile dysfunction medicines, pseudoephedrine, painkillers, antiestrogens, antivirals, hypnotics, antihistamines and anxiolytics. Hundreds of websites were shut down, with seizures worth nearly € 63 million and more than 500 people arrested [18].

The complexity of international supply chains makes it very difficult to trace the origin of a falsified medical product. More sophisticated methods and forensic techniques, such as Isotope Ratio Mass Spectroscopy (IRMS), are needed to get to the bottom of this problem. The development of these techniques can provide solutions for identifying the origin and trade routes of these products, which could in the future be an important tool in combating falsified medicines.

### 1.3 Detection of falsified medicines and vaccines

Many approaches can be used to detect falsified medicines and vaccines, that are summarised in several publications providing an overview of the analytical methodologies employed. [1,16,19–21]

The detection of the falsified medicines/vaccines can be carried out on three different levels: the first one includes the inspection with the naked eye of both packaging and tablets. Level 2 encompasses screening technologies that can be readily completed in the field (or point of sale). The third level involves the techniques that require the equipment of a laboratory to determine medicine quality according to established reference specifications such as pharmacopeial monographs. Usually these include tests for identity, content, dissolution, for related substances/impurities and uniformity of dosage Table 1.

The choice of analytical methods is a balance between available resources and the chemical information sought, in some scenarios (such as low-income countries) the best strategy is not necessarily to have the most specific and precise technique but affordable and simple field detection methods [1].

## 2 Isotopic characterization

Stable isotope ratios have been used as indicators of provenance and source of natural materials since the 1950s when isotope-ratio mass spectrometers were first developed. Sixty-two of the 112 elements are known to have more than one stable isotope, yielding numerous possible isotopic-ratios that may indicate provenance [31]. Stable isotopes have been used for many different purposes including the characterisation of different photosynthetic pathways (C3, C4, CAM), the detection of frauds in food matrices [32], as natural tracers in geology, ecology, archaeology, climatology, plant biology and investigating the illegal wildlife trade. They have also been widely used in proteomics, structural biology, pharmacology and drug design [33].

Carbon.

Carbon is one of the main biogenic elements found in the vast majority of biological molecules. It exists in two stable isotope forms, with δ(^12^C) being the most common with an abundance of 98.93 %, and δ (^13^C) being the least abundant (1.07 %).

Carbon isotope signatures can be used to distinguish plants that use C3 (Calvin cycle), C4 (Hatch-Slack cycle) and CAM (Crassulacean acid metabolism) modes of photosynthesis, as these pathways lead to distinct isotopic fingerprints [34]. A typical C3 plant, such as sugar beet, shows δ13C values ranging from −22 ‰ to −33 ‰. Plants that utilise C4 metabolism (maize, sugarcane and sorghum) demonstrate δ(^13^C) values ranging from −8 ‰ to −16 ‰. Finally, CAM plants including pineapple, cactus, and vanilla, have a metabolism close to C4 plants and similar δ(^13^C) values (−10 ‰ to −16 ‰) [35].

This can be used in the detection of fraud in wine and grapes, as the addition of exogenous sugars (chaptalisation) is illegal in some countries. The supplementation of C4 plant-derived sugars (such as cane sugar) leads to more positive isotopic ratios due to their influence on the δ(^13^C) of ethanol [36].

Carbon isotopes can also be used to reconstruct migratory routes of terrestrial and aquatic animals, in cases where the geographical distribution of C3, C4 and CAM plants is known, as well as the diet preferences of the animal species [37].

Nitrogen.

Nitrogen exists in two stable isotope forms: δ(^14^N) (99.63 %) and δ^15^N (0.37 %). The nitrogen isotopic ratio is expressed with respect to atmospheric nitrogen which has a defined δ(^15^N) value of 0 ‰.

With regard to food authenticity, the nitrogen fingerprint may be used to indicate whether a vegetable is grown with organic or conventional fertilisation methods. The soil-nitrogen is heavily influenced by the type of fertilisers applied and this is reflected in the δ(^15^N) of the plant tissue, which shows more depleted values when the plant is grown with synthetic fertilisers. On the other hand, less depleted values of δ(^15^N) tend to be present in plants grown with animal manures under organic regimes.

Another important application of nitrogen stable isotopes is the identification of an organism’s position in the food chain, or ‘trophic level’ of different species. The heavy nitrogen isotope value (^15^N) increases by (2–4 ‰) when reaching higher trophic levels, through preferential excretion of 14 N during metabolic process, thus serving as a tool in determining dietary shifts [38].

Hydrogen and Oxygen.

Hydrogen and oxygen isotopes have been used extensively as tracers in hydrological, ecological and forensic studies. The spatial variation across the globe, and at the landscape level, of water δ^2^H and δ^18^O is determined by different physical processes and it can be visualised in the mapping of isotopic distributions across the landscape with so-called ‘isoscapes’.

The initial isotopic composition of vapour arriving at the continental margins depends on the initial isotopic composition of water in the vapour-source regions and it is then altered by temperature, affecting rates of evaporation, and humidity. As the air masses go across the continental sites they start losing water due the condensation and precipitation in a temperature-dependent manner. This rainout leads to a progressive depletion of the heavy isotopes in the precipitation resulting in a general decrease of the isotope ratio along trajectories of atmospheric vapour transport and increasing altitude. This effect can be dampened or even completely countered by the vapour coming from the moisture recycling from the continents that is added to the air masses through the hydrological cycle [39].

These processes determine the final isotopic composition of the water available for the plant’s growth, meaning that, knowing how ^2^H and ^18^O isotopes vary for a specific plant, through assimilation and evapotranspiration and photosynthesis, we can infer the origin of the plant from its isotopic composition and relate it to the isotopic composition of the input water in the location where the plant grew.

Stable-isotope measurements.

As per IUPAC (International Union of Pure and Applied Chemistry) protocol, isotopic values are expressed as a delta with respect to the international standard V-PDB (Vienna-Pee Dee Belemnite) for δ(^13^C), V-SMOW (Vienna-Standard Mean Ocean Water) for δ(^2^H) and δ(^18^O), and Air (atmospheric N_2_) for δ(^15^N), following Equation (1):

(1) $$\delta^{i}\left(E_{sample/standard\;}\right)=\frac{R{\left(iE{/}^{j}E\right)}_{sample\;}}{R{\left(iE{/}^{j}E\right)}_{standard\;}}-1$$

Where standard is the international measurement reference material, sample is the analysed sample and iE/jE is the isotope ratio between heavier and lighter isotopes. Delta values are multiplied by 1000 and are commonly expressed in units “per mil” or, according to the International System of Units (SI), as a “milliurey” (mUr).

There are two primary methods for quantifying isotope ratios in the elements that make up organic matter [40]. The first one is isotope ratio measured by mass spectrometry (IRMS), and involves the analysis of isotopic composition by using a mass spectrometer, which separates isotopes based on their mass-to-charge ratio. It involves the full combustion, or thermochemical conversion, of sample molecules and is frequently employed to ascertain the isotopic ratio of bulk substances or the individual components in mixtures when coupled with gas chromatography (GC) or liquid chromatography (LC).

The second technique is SNIF-NMR (site-specific natural isotope fractionation measured by nuclear magnetic resonance) which includes the intra-molecular site-specific analysis of natural isotope fractionation. This can be advantageous for testing the origin of commercial products [40], and can potentially be applied in a similar way to pharmaceuticals.

The aim of the present review is to carry out a comparison of the main isotope-techniques and their implementation in the detection of falsified medicines, with a particular focus on the insights that stable isotope analysis can give about the origin of raw materials used in their production, and the synthetic procedures employed.

### 2.1 Elemental analyzer (EA)/high temperature conversion elemental analyzer (TC/EA) isotope-ratio mass spectrometry (IRMS)

**Fig. 2:**
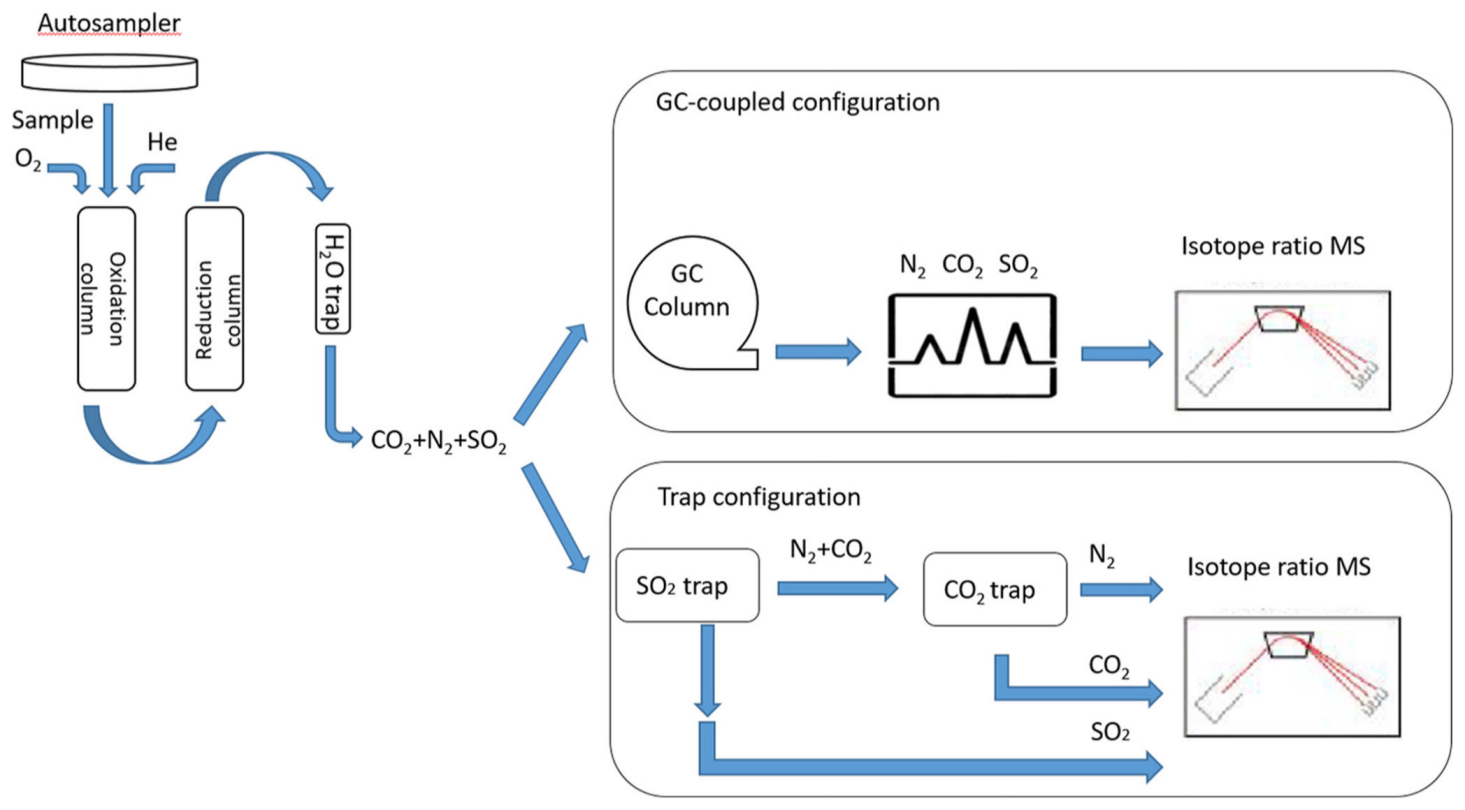
Schematic of an Elemental Analyzer/Isotope Ratio Mass Spectrometer (order of elution from GC column or traps is N_2_, CO_2_ and finally SO_2_).

In EA-IRMS Fig. 2, the analytes (carried by Helium flow) are usually introduced in the system by an autosampler and then combusted quantitatively over metal-oxidecatalysts held at approximately 1000°C, generally with excess of O_2_ added, to produce CO_2_, H_2_O, a mixture of NO_x_ gases and SO_x_. The flow stream runs through a second reactor containing a metal reductant (usually copper at 600°C) to convert NO_x_ to N_2_, SO_x_ to SO_2_, and through a water trap to remove H_2_O. Values of δ(^15^N), *δ*(^13^C), and δ(^34^S) are acquired from the resulting pure N_2,_ CO_2_, and SO_2_ gases respectively, which have been separated either chromatographically or by a trap-and-purge system before isotopic analysis.

**Fig. 3:**
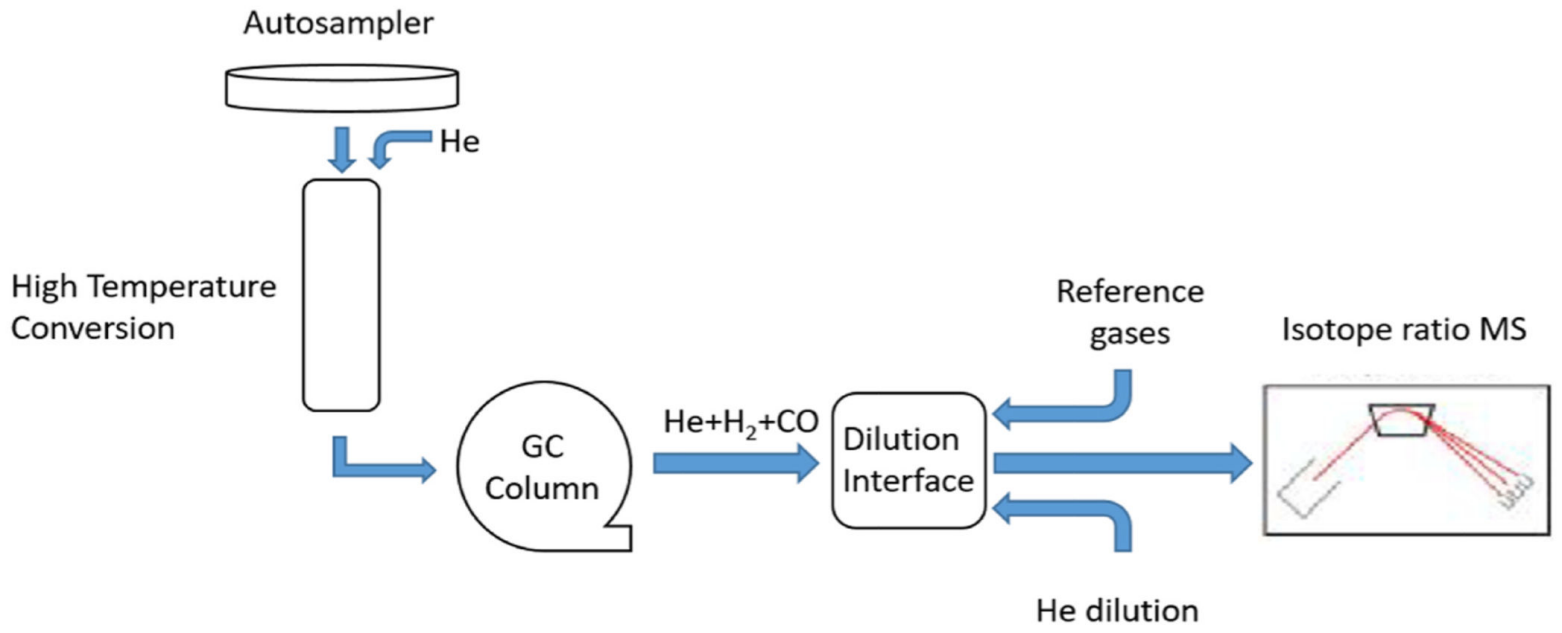
Schematic of a thermal conversion Elementar Analyzer/isotope ratio mass spectrometer.

In TC/EA-IRMS Fig. 3, unlike in the case of EA-IRMS, the oxidation column is not required as the samples are thermochemically converted, usually at temperatures >1400 °C, generating CO for analysis of *δ*(^18^O) and H_2_ for analysis of *δ*(^2^H).

Both configurations are typically connected to the IRMS through a sample/reference dilution device which allows the reference gas and the dilution gas to enter the analysis stream.

After pyrolysis, the gas samples are accelerated into a calibrated set of Faraday cup detectors.

Gas molecules are separated based on their mass to charge ratio by an electro-magnet, with higher ratios corresponding to wider radius travelled by the analytes. The mass distribution of the sample gas is determined relative to the reference gas to obtain an initial ***δ***-value. This initial value is normalised to the internationally accepted ***δ***-scale by the measurement of appropriate standards to carry out a two-point scale calibration.

These two set-ups comprise the so-called bulk analysis, where the isotope value is measured globally and not on single compounds like in other techniques (i.e., GC-C-IRMS).

### 2.2 Gas chromatography - isotope-ratio mass spectrometry (GC-IRMS)

**Fig. 4:**
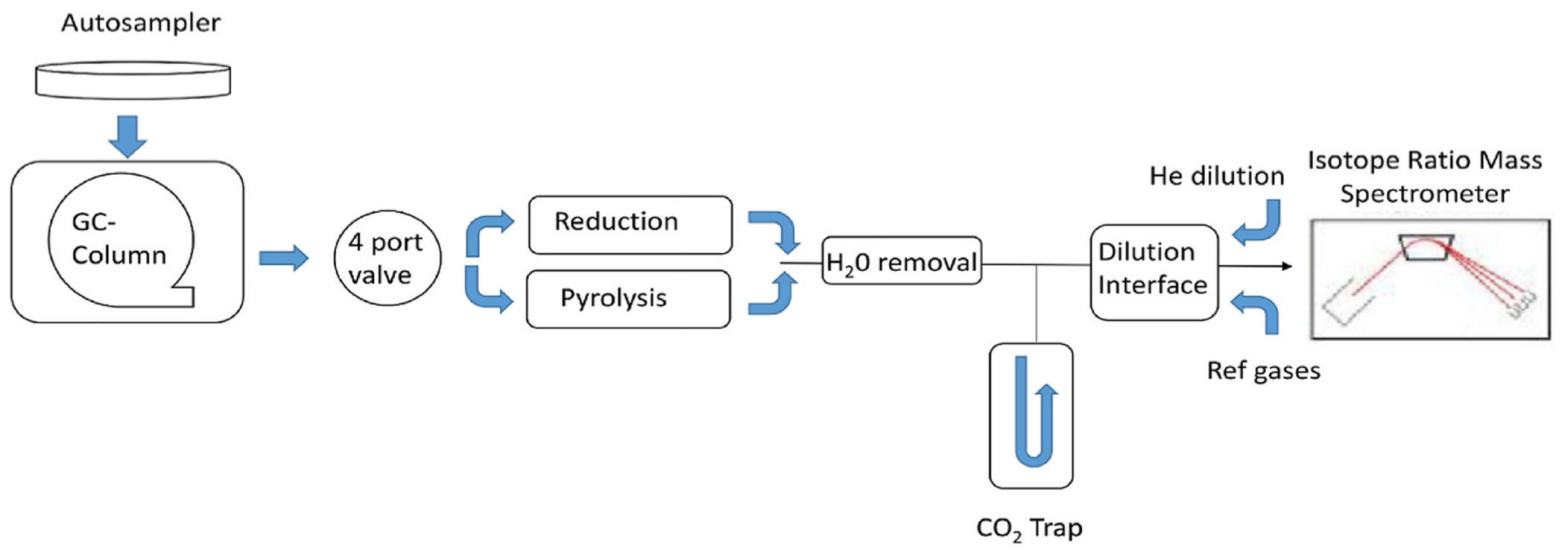
Gas chromatography/isotope ratio-mass spectrometry.

When complex mixtures need to be isotopically analysed, IRMS can be coupled to a gas chromatograph via a combustion interface (GC/C/IRMS) for the analysis of *δ*(^13^C) and *δ*(^15^N), or pyrolysis (GC/Pyr/IRMS) for the analysis of *δ* (^18^O), and *δ*(^2^H). In these cases, analytes are separated through a GC column and then combusted (C/N) or pyrolyzed (H/O) to the corresponding gas (CO_2_, N_2_, H_2_, CO), respectively. Thereafter, the analyte gases pass through a Nafion membrane which removes H_2_O, and a liquid N_2_ trap which removes CO_2_ before entering the IRMS, when *δ*(^15^N) is being determined, because CO2 can crack (or fragment) to CO in the ion-source causing and isobaric interference at *m*/*z* 28 (^14^N^14^N versus interference ^12^C^16^O) Fig. 4.

The primary condition for GC/C/IRMS analysis is that the compounds constituting the sample mixture are volatile and thermally stable. Some non-volatile compounds may also be analysed by this method, requiring further modification (derivatization) in order to make them volatile. In this case, the influence (isotopic composition) of the derivatizating agents must be taken into account.

GC-IRMS has been widely used for the characterization of seized drugs of abuse providing an isotopic “fingerprint” and allowing individual tablets to be linked to a common batch [41,42], but the use of this technique for pharmaceutical products remains limited to a few examples (antidoping test and abuse drugs characterization) [41,43].

### 2.3 Stable-isotope and pharmaceutical medicine tracing

In recent decades, chemical characterization of medicine composition has often been found insufficient for the detection of falsification, as the falsified products, and the techniques used to produce them, have become more sophisticated, with falsified medicines containing the same compounds and having the same elemental composition as the genuine tablets [44].

#### 2.3.1 Active pharmaceutical ingredient (API)

Typical solid dose forms of a medicine contain at least one active pharmaceutical ingredient (API) and a range of excipients of varying quantities. An excipient is a substance that is used alongside the API in the manufacture of medicinal products and can fulfil various functions, such as stabilisation or bulking up the formulation as well as improving therapeutic properties of the API. In addition, excipients are of crucial importance for the manufacturing process, e.g. for handling the API, the flowability of the powder and the compression of the tablets. The synthetic pathway employed and the isotopic ratio of the raw materials determine the isotopic ratio of a specific compound in a pharmaceutical product.

Several chemical processes are needed to render an active ingredient bioavailable and ingestible for the patient. Ibuprofen, a widespread non-steroidal anti-inflammatory (NSAID), is such an example, as it can be synthesised following two different synthetic pathways, which in turn leave different isotopic-fingerprints on the final product [45]. Another important factor that influences the isotope ratio is the selection of the chemical raw materials employed in the synthesis of the API.

#### 2.3.2 Excipients

In cases where falsified medicines contain the same API concentration as the original genuine product, they could differ in the excipient composition [46]. This underlines the need to develop stable isotope methods to study not only the APIs, but also the excipients. An important factor is that many excipients used in pharmaceutical manufacturing are derived from plant (starch, cellulose), animal (lactose) or geological (carbonates) sources. Therefore, they are expected to exhibit isotopic signatures consistent with those different sources [47]. This could be used as a powerful means to trace back to the geographical origin of falsified medicines and vaccines. Only a few papers have been published specifically regarding the stable isotope analysis of excipients.

A multi-faceted approach combining chemical, biological, packaging and palynological data was followed by Newton et al. [48] including the use of IRMS for the mineral composition analysis of artesunate tablets, normally used for malaria treatment, and ultimately achieving the detection of falsified medicines in Southeast Asia. The authors noted that the majority of Vietnam samples contained calcite, which exhibited δ(^18^O) and δ(^13^C) values in a similar range (11‰ to +2‰ and −2‰ to −25‰ respectively), indicating high temperature hydrothermal calcite, mined in Southern China. Palynological evidence combined with the excipient composition data suggested that falsified medicine originated from the border between southern China and Southeast Asia. The evidence presented in this study led to a criminal investigation in the region, which resulted in an arrest of the people responsible for the fraud who had sold hundreds of thousands of falsified artesunate blister packs.

The potential of IRMS as a tool for the traceability of falsified anti-malarials was recently examined by Chesson et al. [49], through the analysis of δ(^13^C) and δ(^18^O) of the starch present in the tablets. Falsified medicines were found to exhibit higher δ(^13^C) values compared to the genuine ones, indicating the addition of maize (C4 plant) -derived starch. On the other hand the authors suggested further investigations for the interpretation of the oxygen data with the purpose of geographical traceability.

Wang et al. [50]investigated the opportunity to trace back the origin of lactose present in pharmaceutical products through stable isotope analysis. δ^13^C analysis of lactose could give hints about the diet of the animal from which the lactose was obtained (C3 based diet vs C4 based diet). On the other hand, H and O values are affected by the water taken by the animal, which therefore, can indicate their geographical origin through direct comparison with a database of excipient materials of known origins. Alternatively, the isotope value of the excipient can be correlated with the Global Meteoric Water Line (GMWL) that provides δ(^18^O) and δ(^2^H) data of precipitation from locations around the globe.

Thirty-four lactose samples from 7 manufacturers from different parts of the world were analysed and the δ(^13^C), δ(^2^H), and δ(^18^O) values of the samples permitted all the lactose samples to be 100% correctly classified according to their manufacturing origin.

Moreover, 3 of the initial 34 lactose samples were used to manufacture 3 drug products. After the lactose extraction the manufacturers of lactose used in the three drug products were correctly identified with 97.06% accuracy. Although no direct information about the geographical origin of lactose was provided in this study, a manufacturer (manufacturing origin) based discrimination was achieved. In addition, the authors proposed a method for lactose extraction that could be used for further traceability studies of such products.

## 3 Trace the origin (IRMS)

### 3.1 Batch to batch and manufactures discrimination

Among the first applications of isotope analysis to pharmaceutical products, was a study carried out by Jasper et al. [51], to determine whether IRMS could distinguish between (i) APIs produced by different manufacturers and (ii) different batches of the same API produced by the same manufacturer. A set of 20 anonymised samples each representing four APIs, namely tropicamide, hydrocortisone, quinine HCl, and tryptophan, was used in this study. These originated from five production batches, manufactured by five different companies. Initially, only the chemical identity of the APIs was revealed to the research team, without any additional information. Based on the chemical composition of each API, the isotopic ratios (^13^C/^12^C, ^15^N/^14^N, ^18^O/^16^O, and/or ^2^H/^1^H) were measured.

The isotopic provenance of the four APIs was determined from bivariate plots of selected isotope ratios, particularly *δ*(^2^H) versus *δ*(^18^O). The authors noted that the multi-isotopic profile of any given API was so specific that it was virtually impossible to precisely (e.g., ±1σ) reproduce it, yielding a high degree of stable-isotopic product authenticity. There is broad consensus in the pharmaceutical community that it would cost more to reproduce a specific multi-isotope profile than to acquire the pharmaceutical product legally. In conclusion, the authors suggested that pharmaceutical firms and other manufacturers could take advantage of pre-measured isotopic composition in raw materials and synthetic intermediates to substantially predetermine the isotopic profile of their pharmaceutical and other products.

Fernandez et al. [52] combined EA-IRMS and MC-ICP-MS to distinguish batches of original antiviral (Heptodin) from falsified medicines. Specifically, they demonstrated how the combination of S, C, N, Mg stable isotope ratios facilitates a strong characterization and can provide evidence even in a forensic investigation.

A different approach was suggested by Felton et al. [53], who applied the concept of pre-measured stable isotope-labelled products in order to verify whether the use of labelled-materials is a useful tool for the batch identification and the detection of falsified products. In order to avoid possible alterations of the API performance, the authors proceeded to label the excipient through unique values of *δ*(^2^H) and *δ*(^13^C)-glucose. This was a practical approach, providing each batch of drug products with different isotopic fingerprints to create a batch-specific identity. Moreover, the authors suggested that combining pre-determined *δ*(^2^H) with *δ*(^13^C), could strengthen the discrimination power and thus enhance the usefulness of this technique in batch identification. It is worth noting that the increased cost induced by the labelling process, was calculated to be 0.8 percent/tablet produced.

### 3.2 Geographical origin

The assessment of the geographical origin of an API based on stable-isotopic fingerprints has shown promising results thus far. However, there are important challenges still to be addressed. Jasper and Gilevska [31,44] aimed to determine the origin of the raw materials of 2 different APIs (topiramate and ibuprofen) using IRMS.

They hypothesised that if the water used in the synthesis of the APIs derived from meteoric water (such as in case of ibuprofen), the sample *δ*(^2^H) and *δ*(^18^O) values could correlate to those of the Global Meteoric Water Line (GMWL). Moreover, Jasper found a linear correlation between the ^13^C values and the hydrogen and oxygen values of Topiramate samples, showing that a blend of carbon sources (C3 and C4 plants) was utilized in the creation of the batches under examination. Even though this was a promising strategy, as noted by Gilevska, it has some drawbacks. Specifically, despite the possibility to compare the oxygen isotope composition *δ*(^18^O) of the annual precipitation in a geographical location to the one measured in the active substance, the oxygen composition of river water may differ from precipitation, especially in dry climate areas.

Wokovic et al. [47] investigated the potential of IRMS analysis in distinguishing the provenance of the APIs Naproxen by testing six differently manufactured products. The great majority of the isotope ratios (*δ*(^18^O), *δ*(^2^H),*δ*(^13^C)) from the six manufacturer’s products lay within relatively well-defined areas or “clusters”. However, the data obtained did not show evidence of geographic provenance, with the authors stating that IRMS analysis can be a plausible means of screening for manufacturer-based isotopic traceability.

Targeting the API for the geographical traceability of pharmaceutical products is definitely a promising approach, however further studies are needed to understand the underlying theoretical basis for such a discrimination of origin. As noted by Jasper, further research that considers the isotopic values of starting materials and final products would allow for the determination of the isotope fractionation factors of the synthetic reactions.

### 3.3 Synthetic pathway

As discussed above, the isotopic profile of a drug is a function of both raw materials and the synthetic pathway employed in its production [31]. However, when the isotope values of the starting materials are unknown, the effect of the kinetic fractionation occurring during synthesis on the measured values cannot be quantified with certainty.

Gilevska et al. [44] aimed to identify which of the two most common synthetic pathways (the Darzan route or the carbonylation route) were used to produce 28 commercial ibuprofen samples (API obtained by solvent extraction) and 4 pure APIs, through multidimensional isotope analysis (*δ*(^2^H), *δ*(^13^C),*δ*(^18^O)). Although 5 groups could be differentiated according to the manufacturer, the elucidation of the chemical process remained speculative due to the fact that the producers were not willing to disclose their production lines, or the raw materials used. The depletion in the isotope composition of hydrogen was attributed to 2 manufacturer groups following the carbonylation synthetic route, in accordance with Deconinck [54], while 2 other groups followed Darzan’s route due to the enriched isotopic value of hydrogen. The last group could not be clearly related to a synthetic pathway.

**Fig. 5:**
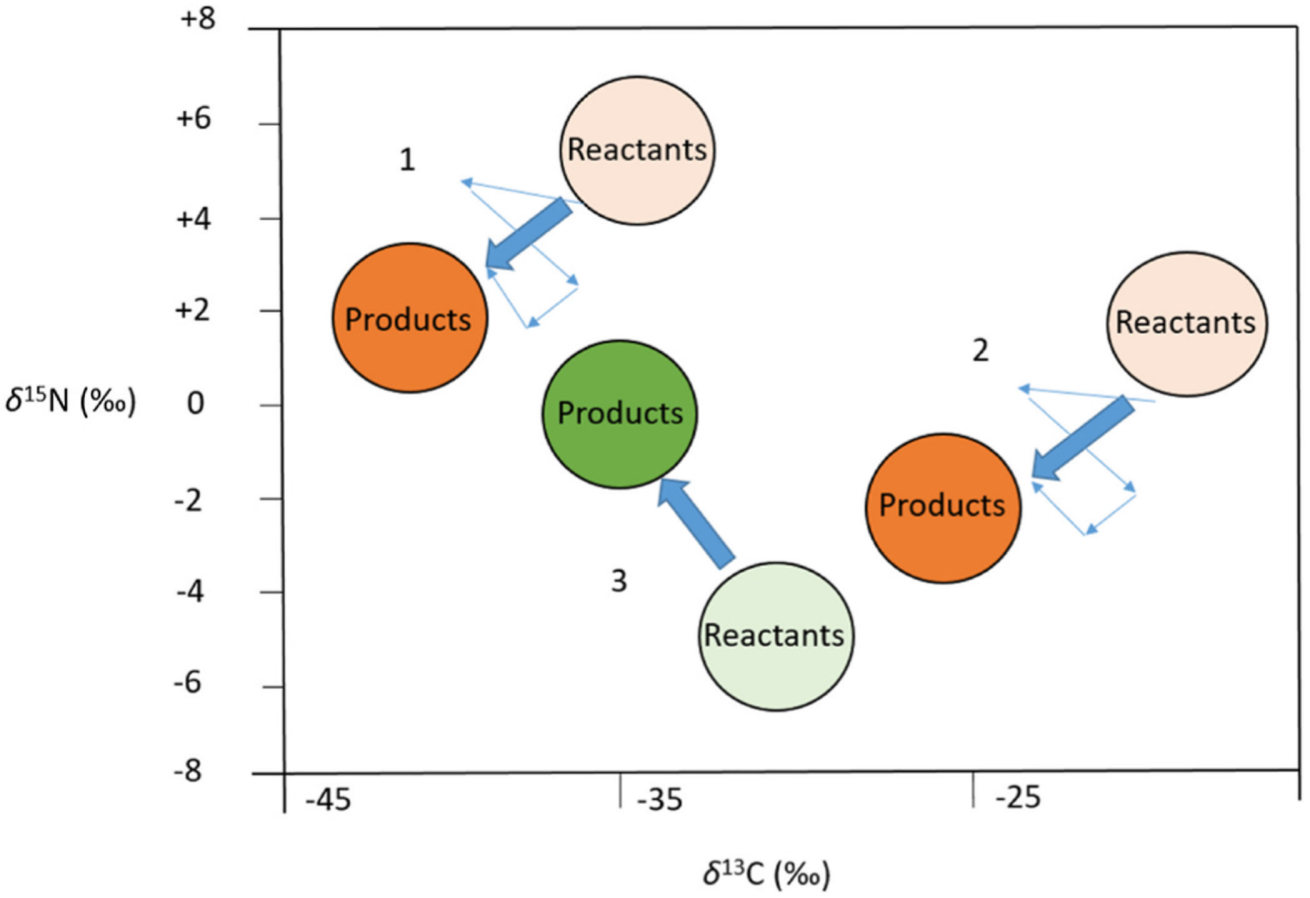
A bivariate plot of nitrogen (δ^15^N) and carbon (δ^13^C) isotopic results for a small molecule antibiotic produced by an authentic pathway (scheme 1), by an identical, and therefore infringing, generic pathway (scheme 2), and by a noninfringing generic pathway (scheme 3) [55].

In IRMS analysis it is critical to know the isotope values of the raw materials in order to understand which chemical procedures were followed in the production of a specific drug. This principle was applied in the resolution of a patent infringement case [55], where a company was accused of selling a generic version of an antibiotic drug. This was suspected to have been produced following the plaintiff’s patented manufacturing process. The synthetic procedure was known to exhibit a predictable fractionation pattern for both C and N. The measurements of the isotopic values of the reactants and products were translated in a bivariate plot (*δ*(^13^C) vs *δ*(^15^N)) to a vector of known direction and magnitude Fig. 5 (the vector was a sum of all individual fractionation processes of the reaction intermediates). The extent and orientation of the vector indicated that the defendant was employing the same synthetic process rather than using an alternative synthesis. Different manufacturers with different suppliers may have different isotopic values of the raw materials, however, the net isotopic vector remains the same if the same synthetic pathway is employed. This work showcased the applicability of IRMS analysis in assessing the synthetic pathway used.

## 4 Nuclear magnetic resonance (NMR)

Stable-isotope analysis can also be carried out using quantitative NMR spectroscopy. This technique offers site-specific (or intra-molecular) information as to the stable isotope content at natural abundance and allows, in principle, the evaluation of the isotopic value (*δ*(^2^H) or *δ*(^13^C)) at each atom of the molecule. Often, only a few key positions of a molecule are taking part in the reaction mechanism, and only the corresponding *δ*(^2^H) and *δ*(^13^C) ratios are affected [45]. By using IRMS, the information on a single position can get lost when overall *δ*(^2^H) and *δ*(^13^C) values are measured. Measuring the site-specific isotope ratios can give suggestions on the specific reactions employed in a synthetic procedure, and not simply help in identifying the manufacturer [45]. Recently, this technique has been applied to drugs of abuse such as methamphetamine [56] and pharmaceuticals, specifically fluoxetine [57].

### 4.1 SNIF-NMR

Site-Specific Natural Isotope Fractionation measured by Nuclear Magnetic Resonance (^2^H–SNIF-NMR) is a well-established technique, developed in the early 1980’s by Martin & Martin [58], widely employed in food authentication for the official control of wine [59], spirits [60], flavours and fruit juices [61]. This method has also been applied to pharmaceutical products by Acetti et al. [45]. The authors recorded the deuterium NMR spectra of two common drugs, naproxen and ibuprofen, in order to obtain information about the starting materials and the synthetic procedure employed.

#### 4.1.1 Naproxen

**Fig. 6:**
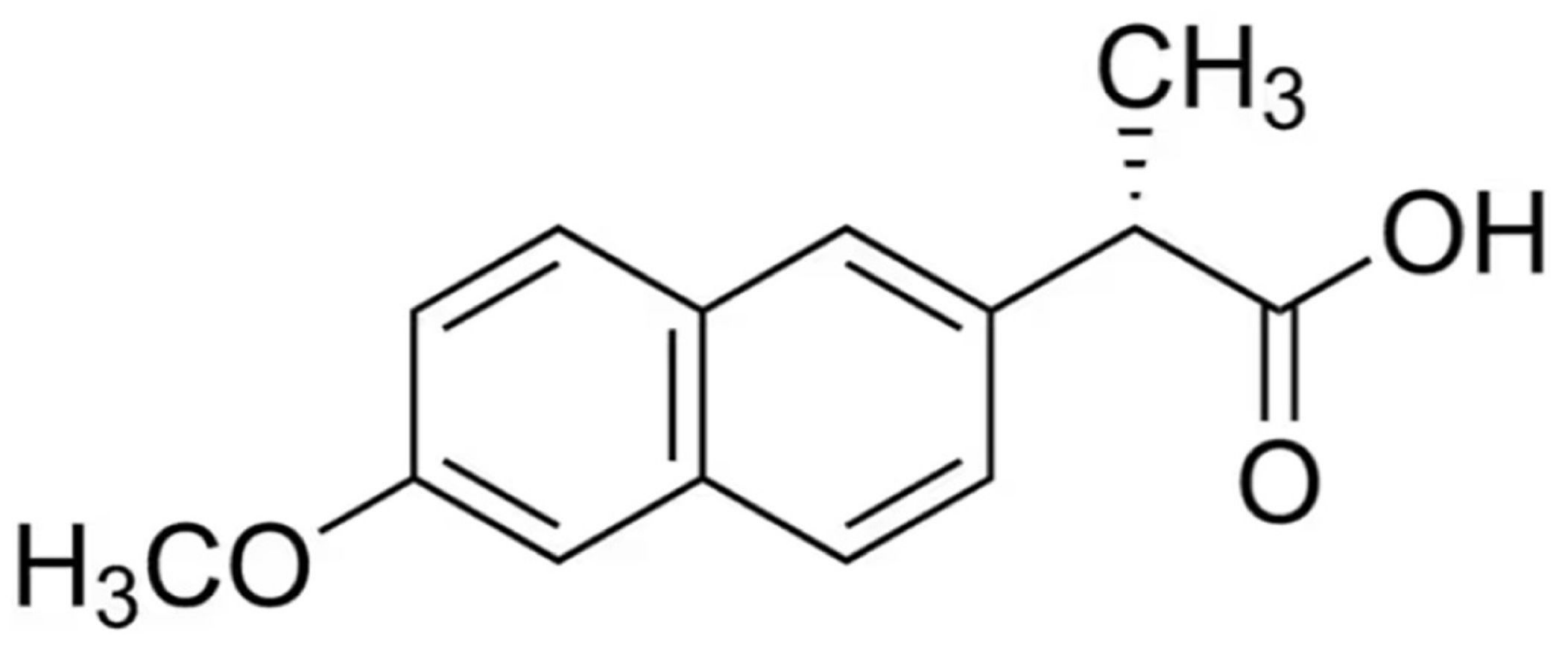
Naproxen molecule.

Naproxen is a nonsteroidal anti-inflammatory drug (NSAID) widely used and manufactured worldwide [45] Fig. 6. Only one procedure is mainly used to manufacture this molecule and it employs 2 possible starting materials. The authors recorded the deuterium spectra of each hydrogen with the purpose of obtaining hints on procedures employed.

The *δ*(^2^H) values of the various positions of naproxen methyl ester show that the most significant data are those of the methoxy group (OCH_3_) of the naphthyl moiety. According to the authors the *δ*(^2^H) values of OCH_3_ clearly resemble the two possible origins of the methoxy group. Four of the 5 samples showed similar *δ*(^2^H) values (ranging from 142.1 ppm to 155.0 ppm) and seem to come from a common naphthyl starting material (when β -naphthol is used, the methyl group is inserted by reaction with methyl chloride in basic medium, and methyl chloride is produced by chlorination of methane). On the other hand, the higher deuterium content (189.4 ppm) of the OCH_3_ group of the remaining sample seems to suggest the synthesis via a different naphthyl precursor (2-Methoxynaphthalene is indeed prepared by reaction of β -naphthol with dimethyl sulphate so that the methyl group derives from methanol, which comes ultimately from methane oxidation). However, other studies could not derive useful information by using ^2^H NMR even when the naproxen samples were converted to the corresponding methyl ester [62].

#### 4.1.2 Ibuprofen

**Fig. 7:**
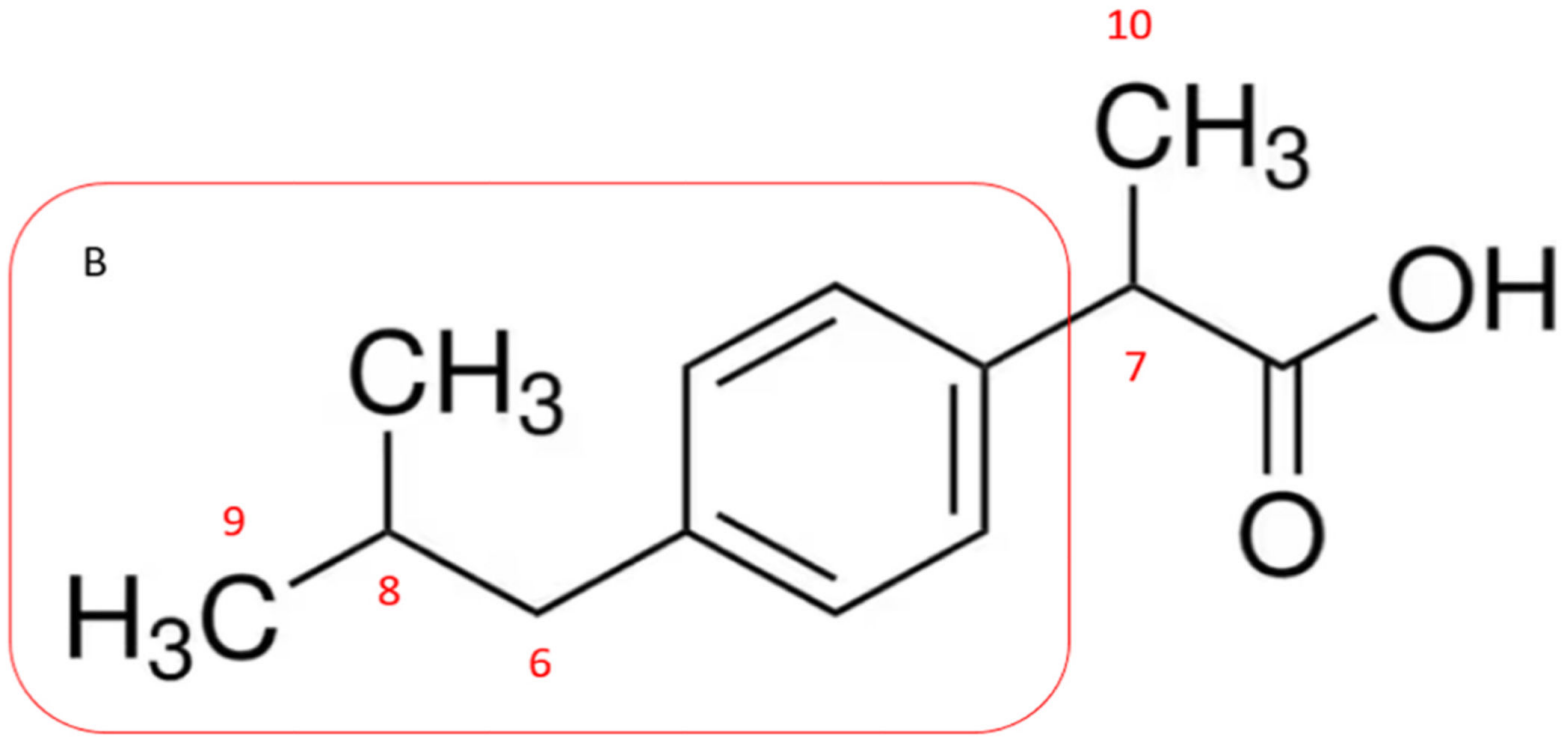
Ibuprofen numbered carbon.

Ibuprofen Fig. 7 is another commonly used NSAID, with the two main synthetic pathways for production being the Boots process and the BHC process. Both of these routes employ isobutylbenzene as a starting material. In the Boots process, the acylation of isobutylbenzene is firstly employed, followed by the Darzens reaction of the ketone to obtain the ester. Thereafter, hydrolysis and decarboxylation are performed to obtain the aldehyde which is converted to the corresponding acid through the last oxidation step. In the BHC process, acylation of isobutylbenzene is done in the presence of hydrogen fluoride by acetic anhydride followed by conversion into alcohol by hydrogen reduction in the presence of a raney-nickel catalyst; the hydroxyl group then reacts with carbon monoxide in the presence of a palladium catalyst to obtain ibuprofen.

Acetti et al. [45] noted that the analysis of the δ(^2^H) value of the aromatic hydrogens and those belonging to the CH_3_ group shows no great variation. Conversely, ibuprofen samples could be grouped in 3 clusters based on the δ(^2^H) value of the CH-7. Therefore, the authors speculated that, given the high deuterium content (mean value 166 ppm), 1 group was prepared through the Darzans and oxime route while the second group (116 ppm) seemed to come from the hydrogenation and carbonylation sequence. This is in line with the fact that hydrogen atoms generally show a low deuterium content when they are inserted by means of molecular hydrogen [45]. The third group (represented by 1 sample) showed a surprisingly high δ(^2^H) (CH) value probably due to isotope effects connected with the oxidation of the aldehydic moiety in a basic medium.

These considerations were sustained by the analysis of the δ(^2^H) values of alcohol prepared from isobutylacetophenone according to three different reduction procedures.

Many data have been collected by different authors on ibuprofen molecules: Remaud et al. [62] similarly concluded that the δ(^2^H) value of the CH-7 group is heavily affected by the synthetic pathway and it allows the discrimination of the 2 main routes employed in the synthesis.

According to the results obtained so far in this work ^2^H SNIF-NMR appears to be a useful tool to trace back the synthetic procedure for the production of ibuprofen whereas it has not reached the same outcomes for the traceability of starting materials employed for the production of naproxen. Nevertheless, some limitations have emerged, including the low sensitivity of the deuterium probe, the potential H-exchange with the solvent during the manufacturing process and the lack of resolution due to overlapping signals. Furthermore, the applications of ^2^H NMR are limited to small molecules (250–300 g/mol-1) [62].

In an attempt to overcome these difficulties, scientists developed a new technique named quantitative isotopic ^13^C NMR spectrometry at natural abundance, allowing the measurement of the ^13^C content of each carbon within a molecule. Remaud and Bussy [40,62] also recorded the δ(^13^C) spectrum of the different C positions of the ibuprofen API: the part B of the molecule in (Fig. 7) is the common synthon for both pathways so the information about the origin of the starting materials should lie within this portion of the molecule.

Specifically, carbon 4 and 5 show the lowest variation as they come from petrochemical crude oil, carbon 9 seems to be influenced by the differences in the raw materials used in each geographic region. Within this portion of the molecule the largest variation is observed on carbon 6 which is the carbon where the addition of the aromatic ring occurs, therefore a large set of isotopic profiles could be displayed as different reagents and reactions can be employed to create the bond.

The 2 main routes differ from one another by the building of the acetate side chain [40], therefore the C-7 carbon, which is involved in the addition of the acetate group, potentially represents a significant part of the information for the isotopic discrimination of samples according to their synthetic pathways. C-10 gives information on the origin of the methyl group of the ‘acetate’ moiety.

Caytan et al. [63] and Thibaudeau et al. [64] investigated the performance of ^13^C NMR with a particular focus on the 2 main weaknesses of this technique: poor sensitivity and long experimental time, ultimately proposing some adjustments to overcome these.

#### 4.1.3 Acetylsalicylic acid and Parcetamol

**Fig. 8:**
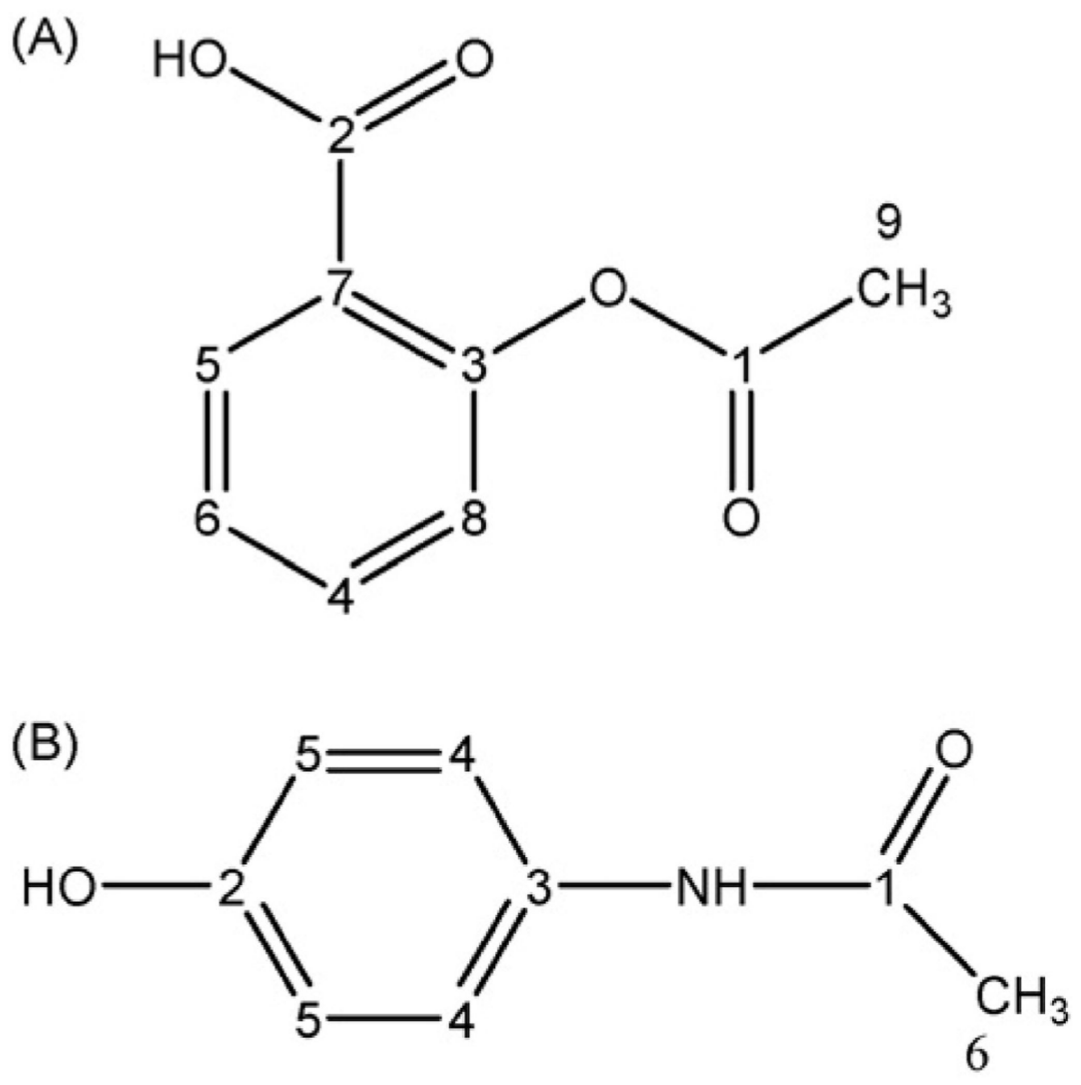
Molecular structures of (A) acetylsalicylic acid and (B) paracetamol with carbon positions numbered in order of decreasing ^13^C chemical shift.

^13^C NMR has also been applied by Silvestre et al. [65] to two commonly used medicines available world-wide: aspirin and paracetamol. The APIs of these medicines are acetylsalicylic acid and paracetamol (acetaminophen), respectively. Fig. 8 They measured the global δ(^13^C) value by IRMS and the site-specific δ(^13^C) value by NMR.

As noted earlier, only some positions of the APIs show wide variations in the δ(^13^C), in particular C-1 C-2 and C-9 for acetylsalicylic acid and C-1 C-3 and C-6 for acetaminophen acid, whereas the remaining carbons have standard deviations similar to or lower than the global value. Even considering only the δ(^13^C) value of each aromatic carbon, each sample is individually characterised. Nevertheless, the main difference between samples for both API seems to lie in the side chains. In particular, the portion of the molecule which shows the largest variation is the acetyl group added during synthesis. Hence, the authors suggested that the isotopic ratio of the carbon in the substituent group could be used to discriminate between batches. On the other hand, the δ(^13^C) composition of the aromatic ring indicated consistency in the raw material, which was coherent with the likely utilisation of phenol as starting material for both compounds.

The authors also speculated on the origin of the acetic acid employed to build the side chain distinguishing the provenance according to the δ(^13^C) value. Some samples, having very negative values (≃-50 δ(^13^C)), seemed to have a petrochemical origin whereas for others samples with less negative values (≃ −17 δ(^13^C)), suggesting a natural origin.

In conclusion, the authors demonstrated that the quantitative isotopic ^13^C NMR is a promising method to obtain an individual isotopic fingerprint for pharmaceuticals. This allowed for accurate discrimination between batches that can be included in the arsenal of isotopic techniques available to trace the orgins of falsified medicines.

## 5 Conclusion

In this article we examined the applicability of stable isotope ratio methods for tracing the origin of falsified medicines. The number of articles about this topic is limited, since it is an emerging approach, but interesting feasibility studies have been reported in the literature. IRMS proved to be a powerful tool for discriminating between different batches of drug production. In such cases, this technique employs quick sample preparation procedures, it is fairly fast and shows strong discriminating power, especially when more than 1 isotopic ratio is considered. In future, this could also be used as a tool to classify a product as falsified if the “criminal intent” cannot be proven by any other method.

However, the issue is more challenging and nuanced when it comes to identifying the geographic origin and thus the origin of falsified products and/or their components. A comparison of the H and O values of the API with the GMWL could provide insights about the origin of the products. However, the APIs are synthetically produced molecules and therefore they usually undergo several chemical reactions that can heavily affect the isotopic ratios, which in turn confounds isotopic interpretation. Consequently, the geographic information can be lost.

A possible solution in overcoming this problem, would be analysing the water entrained in the falsified medicine during production, since it is likely to have the same origin as the location where the medicine was manufactured. However, this presents other technological challenges in quantitatively recovering and measuring very small quantities of water without introducing isotopic fractionation. Furthermore, storage conditions could impact on the long-term integrity of the water’s isotopic signature.

Another promising strategy suggested for tracing the geographical origin of falsified medicine was the analysis of the excipients, since many excipients (such as starch, lactose and cellulose) are plant or animal derived materials that exhibit characteristic hydrogen and oxygen isotope signals related to their place of cultivation/production.

Lastly, the creation of reference datasets of isotopic values of excipients with known provenance is invaluable for the comparison of suspect sample experimental values. The limiting factor will be finding a representative amount of samples with known provenance, which may be expensive and time consuming. However, isotopic datasets of different plants (such as cotton) are already available, which report isotopic values of major production areas. These datasets could be used to estimate isotopic fractionation along geographical coordinates for related agricultural products such as cellulose and starch once the isotopic correlation between different plant materials and components has been established by baseline experiments.

There has been little public domain research on use of IRMS techniques to determine the origin of falsified pharmaceuticals with more progress in related fields, such as food fraud and the illegal wildlife trade. As there are existing networks of specialised laboratories for such investigations, they could potentially be also used for the neglected problem of countering falsified medicines, along with other related techniques such as packaging and environmental DNA analysis [66]. Care will be needed in the publication of results to avoid giving information that would enable criminals to avoid such novel forensic detection.

## Figures and Tables

**Table 1:** Overview of devices for the detection of SF medicines in supply chains. See Ref. [16] for more information.

Method		Description		Advantages		Disadvantages
Level 1						
Visual inspection/ bulk testing		A combination of techniques based on the comparison of the suspected medicine with a genuine one [1]. (printing, embossing, shape, odour, taste, consistency, wt, density, refractive index, viscosity, osmolarity, pH, crystal morphology, solubility).		Inexpensive and rapid		Requires comparator genuine samples [21]
Level 2						
Spectroscopic portable techniques		Based on the irradiation of a sample with specific wavelengths, the energy absorbed causes the vibration of the chemical structures of the sample that can be measured by NIR, MIR, Raman spectroscopy, UV-vis [19].		- Rapid - Relatively Inexpensive compared to laboratory devices		- Requires the creation of a referenceg spectra dataset [16] Usually not quantitative
Thin layer chromatography (TLC)		Widely used in LMICs (low and middle income countries), consisting essentially of a chemical separation of the product’s component which takes place on a silica layer followed by visual detection of the colour spots or use of a UV lamp [1].		- Fast - Convenient - Specific - Inexpensive - Ease of use		- Often uses toxic or flammable reagents - Similar chemical structures may cause similar distance imprecision [22] Not quantitative or semiquantitative
Colorimetry		Identifies particular active ingredients by making use of colour changes produced by chemicals [1]. Used as a pass/fail test or in semi-quantitative mode [23]		- Rapid - Does not require extensive training		- Less sensitive than other in-lab techniques and not available for many APIs [21] Not quantitative
GPHF Minilab®		Portable kit, which allows the examination of a suspected SF medicine in 3 steps: - visual inspection of dosage form and associated packaging material. disintegration test as surrogate to assess drug solubility semiquantitative TLC test [23,24]		- Reliable and simple - Inexpensive		- Requires training - Less sensitive than other “in-lab” techniques Not quantitative or semi quantitative
Level 3						
Dissolution test		Dissolution testing evaluates the degree and speed at which an API dissolves from a specific dosage form. The dissolution process of a drug is crucial as it directly impacts its bioavailability and therapeutic efficacy [25].		- Can detect inappropriate formulation even when the amount of the API is correct		- Might not fully capture the entire complexity of drug behaviour in vivo.
X-ray diffraction methods		Based on the comparison of the diffraction pattern of genuine and suspected drugs [26].		- Fast and reliable, - Portable - Does not require sample preparation.		- Provides only qualitative analysis - Costly
Conventional and Spatially-offset Raman spectroscopy		Obtaining a spectrum resulting from the medicine components and comparing it with the spectra stored in a database [21].		- No sample preparation - Fast (less than a minute) collection times required - nondestructive[20,23]		- Qualitative analysis and quantitative for some APIs - High initial investment cost
Chromatography techniques: (GC) Gas chromatography, (LC) liquid chromatography, (HPLC)highperformance liquid chromatography, (CE) capillary electrophoresis		Separating mixtures into components based on various chemical and physical properties [27]. The most common analytical method used in medicine evaluations [28].		- High sensitivity - sensitive - accurate - reproducible, quantitative		- Sophisticated - Costly - Labor-intensive - Requires sophisticated equipment and training [16]
Mass spectrometry (MS): such as high-resolution time-of-flight (TOF) MS and MS/MS		Produces a mass spectrum which is a plot of intensity as a function of the mass-to-charge ratio [29]. Accurate identification of ingredients presents in falsified drugs		- Specific - Precise		Expensive and sophisticated lab analysis
Hyphenated techniques		Methods developed from combining separation techniques and an on-line spectroscopic detection technology such as GC/MS LC/MS [30].		- Sensitive - Accurate		- Requires training - Expensive equipment

## Data Availability

No data was used for the research described in the article.

## References

[1] Newton PN, Green MD, Fernández FM, Day NPJ, White NJ (2006). Counterfeit anti-infective drugs. Lancet Infect Dis.

[2] (2017). WHO global surveillance and monitoring system. World Health Organization.

[3] Hagen N, Bizimana T, Kayumba PC, Khuluza F, Heide L (2020). Stability of oxytocin preparations in Malawi and Rwanda: stabilizing effect of chlorobutanol. Am J Trop Med Hyg.

[4] WHO (2017). Member State Mechanism on Substandard/spurious/falsely-Labelled/falsified/Counterfeit Medical Products.

[5] Hauk C, Hagen N, Heide L (2021). Identification of substandard and falsified medicines: influence of different tolerance limits and use of authenticity inquiries. Am J Trop Med Hyg.

[6] World Health Organization (2017). A Study on the Public Health and Socioeconomic Impact of Substandard and Falsified Medical Products.

[7] Schier J, Chang A, Kapil V (2023). Medication-associated diethylene glycol mass poisoning — a preventable cause of illness and death. N Engl J Med.

[8] Cavany S, Nanyonga S, Hauk C, Lim C, Tarning J, Sartorius B (2023). The uncertain role of substandard and falsified medicines in the emergence and spread of antimicrobial resistance. Nat Commun.

[9] World Health Organization (2018). Substandard and falsified medical products.

[10] UNITED NATIONS OFFICE ON DRUGS AND CRIME (2019). COMBATING FALSIFIED MEDICAL PRODUCT-RELATED CR ME A GUIDE TO GOOD LEGISLATIVE PRACTICES, UNITED NATIONS.

[11] Bergström R (2013). Anti-counterfeiting: finding solutions to a global problem.

[12] Antonopoulos AHRK (2017). Illicit pharmaceutical networks in Europe: organising the illicit medicine market in the United Kingdom and The Netherlands. Trends Organ Crime.

[13] OECD Covid-19 crisis underscores need to address trade in fake pharmaceuticals, say OECD & EUIPO. OECD Better policies for better lives.

[14] Cueni TB (2017). The Trade Routes of Counterfeits.

[15] Glass BD (2014). Counterfeit drugs and medical devices in developing countries. Res Rep Trop Med.

[16] Kelesidis T, Kelesidis I, Rafailidis PI, Falagas ME (2007). Counterfeit or substandard antimicrobial drugs: a review of the scientific evidence. J Antimicrob Chemother.

[17] Europol (2015). Situation Report on Counterfeiting in the European Union.

[18] Europol Europol (2021). 544 Arrests and €63 Million of Fake Pharmaceuticals and Illegal Doping Substances Seized.

[19] Vickers S, Bernier M, Zambrzycki S, Fernandez FM, Newton PN, Caillet C (2018). Field detection devices for screening the quality of medicines: a systematic review. BMJ Glob Health.

[20] Deisingh AK (2005). Pharmaceutical counterfeiting. Analyst.

[21] Kelesidis T, Falagas ME (2015). Substandard/counterfeit antimicrobial drugs. Clin Microbiol Rev.

[22] Bolla AS, Patel AR, Priefer R (2020). The silent development of counterfeit medications in developing countries - a systematic review of detection technologies. Int J Pharm.

[23] Fernandez FM, Green MD, Newton PN (2008). Prevalence and detection of counterfeit pharmaceuticals: a mini review. Ind Eng Chem Res.

[24] GPHF The GPHF-MinilabTM - Protection Against Counterfeit Medicines. The Global Pharma Health Fund (GPHF).

[25] What is the USP dissolution test?.

[26] Maurin JK, Pluciński F, Mazurek AP, Fijałek Z (2007). The usefulness of simple X-ray powder diffraction analysis for counterfeit control—the Viagra® example. J Pharmaceut Biomed Anal.

[27] Institute of Medicine (2013). Countering the Problem of Falsified and Substandard Drugs.

[28] Martino R, Malet-Martino M, Gilard V, Balayssac S (2010). Counterfeit drugs: analytical techniques for their identification. Anal Bioanal Chem.

[29] Price P (1991). Phil Price, Standard definitions of terms relating to mass spectrometry A report from the committee on measurements and standards of the American society for mass spectrometry. J Am Soc Mass Spectrom.

[30] Patel KN, Patel JK, Patel MP, Rajput GC, Patel HA (2010). Introduction to hyphenated techniques and their applications in pharmacy. Pharm Methods.

[31] Jasper JP, Weaner LE, Duffy BJ (2005). A preliminary multi-stable-isotopic evaluation of three synthetic pathways of Topiramate. J Pharm Biomed Anal.

[32] (2019). C and H stable isotope ratio analysis using solid-phase microextraction and gas chromatography-isotope ratio mass spectrometry for vanillin authentication. J Chromatogr A.

[33] Zachleder V, Vítová M, Hlavová M, Moudříková Š, Mojzeš P, Heumann H (2018). Stable isotope compounds - production, detection, and application. Biotechnol Adv.

[34] Cernusak LA, Ubierna N, Winter K, Holtum JAM, Marshall JD, Farquhar GD (2013). Environmental and physiological determinants of carbon isotope discrimination in terrestrial plants. New Phytol.

[35] Modern Techniques for Food Authentication.

[36] Perini M, Pianezze S, Guardini K, Allari L, Larcher R (2023). Authentication and geographical characterisation of Italian grape musts through glucose and fructose carbon isotopic ratios determined by LC-IRMS. Molecules.

[37] Zimmo Sahar, Department of biology, university of western ontario, London, ontario, Canada), jake blanco (department of biology, university of western ontario, London, ontario, Canada) & silke nebel (department of biology, university of western ontario, London, ontario, Canada) (2012). The use of stable isotopes in the study of animal migration. Nature Education knowledge.

[38] (2008). Tracking Animal Migration with Stable Isotopes.

[39] Bowen GJ (2008). Spatial analysis of the intra-annual variation of precipitation isotope ratios and its climatological corollaries. J Geophys Res.

[40] Bussy U, Thibaudeau C, Thomas F, Desmurs JR, Jamin E, Remaud GS (2011). Isotopic finger-printing of active pharmaceutical ingredients by 13C NMR and polarization transfer techniques as a tool to fight against counterfeiting. Talanta.

[41] Carter JF, Titterton aEL, Murray M, Sleeman R (2002). Isotopic characterisation of 3,4-methylenedioxyamphetamine and 3,4-methylenedioxymethylamphetamine (ecstasy). Analyst (Cambridge, UK).

[42] Palhol F, Lamoureux C, Naulet N (2003). 15N isotopic analyses: a powerful tool to establish links between seized 3,4-methylenedioxymethamphetamine (MDMA) tablets. Anal Bioanal Chem.

[43] Iannella L, Botré F, Colamonici C, Curcio D, de la Torre X (2019). Development and validation of a method to confirm the exogenous origin of prednisone and prednisolone by GC-C-IRMS. Drug Test Anal.

[44] Gilevska T, Gehre M, Richnow HH (2015). Multidimensional isotope analysis of carbon, hydrogen and oxygen as tool for identification of the origin of ibuprofen. J Pharm Biomed Anal.

[45] Acetti D, Brenna E, Fronza G, Fuganti C (2008). Monitoring the synthetic procedures of commercial drugs by 2H NMR spectroscopy: the case of ibuprofen and naproxen. Talanta.

[46] Pisklak DM, Zielińska-Pisklak MA, Szeleszczuk Ł, Wawer I (2016). 13C solid-state NMR analysis of the most common pharmaceutical excipients used in solid drug formulations, Part I: chemical shifts assignment. J Pharm Biomed Anal.

[47] Wokovich AM, Spencer JA, Westenberger BJ, Buhse LF, Jasper JP (2005). Stable isotopic composition of the active pharmaceutical ingredient (API) naproxen. J Pharm Biomed Anal.

[48] Newton PN, Fernández FM, Plançon A, Mildenhall DC, Green MD, Ziyong L (2008). A collaborative epidemiological investigation into the criminal fake artesunate trade in South East Asia. PLoS Med.

[49] Newton Paul N, Chesson Lesley A, Mayxay Mayfong, Dondorp Arjen, Tabernero Patricia, Howa John D, Cerling Thure E Forensic Investigation of Falsified Antimalarials Using Isotope Ratio Mass Spectrometry – a Pilot Investigation.

[50] Wang Yu-ye, Yang Fan, Chen Jian, Li Ying-jian, Zhou Jia, Yan Dong, Lu Xin, Zhou Peng, Zhang Li (2023). Multidimensional isotope analysis of carbon, hydrogen, and oxygen as a tool for traceability of lactose in drug products. J Pharmaceut Biomed Anal.

[51] Jasper JP, Westenberger BJ, Spencer JA, Buhse LF, Nasr M (2004). Stable isotopic characterization of active pharmaceutical ingredients. J Pharm Biomed Anal.

[52] Santamaria-Fernandez R, Hearn R, Wolff JC (2009). Detection of counterfeit antiviral drug Heptodin and classification of counterfeits using isotope amount ratio measurements by multicollector inductively coupled plasma mass spectrometry (MC-ICPMS) and isotope ratio mass spectrometry (IRMS). Sci Justice.

[53] Felton LA, Shah PP, Sharp Z, Atudorei V, Timmins GS (2011). Stable isotope-labeled excipients for drug product identification and counterfeit detection. Drug Dev Ind Pharm.

[54] Deconinck E, van Nederkassel AM, Stanimirova I, Daszykowski M, Bensaid F, Lees M (2008). Isotopic ratios to detect infringements of patents or proprietary processes of pharmaceuticals: two case studies. J Pharm Biomed Anal.

[55] Sabatelli AD, Pearson A, Jasper JP (2017). Process patent protection via analysis of stable isotope ratios. Org Process Res Dev.

[56] Armellin S, Brenna E, Frigoli S, Fronza G, Fuganti C, Mussida D (2006). Determination of the synthetic origin of methamphetamine samples by 2H NMR spectroscopy. Anal Chem.

[57] Brenna E, Fronza G, Fuganti C (2007). Traceability of synthetic drugs by position-specific deuterium isotope ratio analysis: the case of Prozac and the fluoxetine generics. Anal Chim Acta.

[58] Martin GJ, Martin ML (1981). Deuterium labelling at the natural abundance level as studied by high field quantitative 2H NMR. Tetrahedron Lett.

[59] Solovyev PA, Fauhl-Hassek C, Riedl J, Esslinger S, Bontempo L, Camin F (2021). NMR spectroscopy in wine authentication: an official control perspective. Compr Rev Food Sci Food Saf.

[60] Fotakis C, Zervou M (2016). NMR metabolic fingerprinting and chemometrics driven authentication of Greek grape marc spirits. Food Chem.

[61] Ogrinc N, Bat K, Kosir IJ, Golob T, Kokkinofta R (2009). Characterization of commercial slovenian and cypriot fruit juices using stable isotopes. J Agric Food Chem.

[62] Remaud GS, Bussy U, Lees M, Thomas F, Desmurs JR, Jamin E (2013). NMR spectrometry isotopic fingerprinting: a tool for the manufacturer for tracking active pharmaceutical ingredients from starting materials to final medicines. Eur J Pharmaceut Sci.

[63] (2007). Precise and accurate quantitative 13C NMR with reduced experimental time. Talanta.

[64] Thibaudeau C, Remaud G, Silvestre V, Akoka S (2010). Performance evaluation of quantitative adiabatic 13C NMR pulse sequences for site-specific isotopic measurements. Anal Chem.

[65] Silvestre V, Mboula VM, Jouitteau C, Akoka S, Robins RJ, Remaud GS (2009). Isotopic 13C NMR spectrometry to assess counterfeiting of active pharmaceutical ingredients: site-specific 13C content of aspirin and paracetamol. J Pharm Biomed Anal.

[66] Young JM, Liddicoat C, van Dijk KJ, Tabernero P, Caillet C, White NJ (2022). Environmental DNA as an innovative technique to identify the origins of falsified antimalarial tablets—a pilot study of the pharmabiome. Sci Rep.

